# Supplementary figures and images for: Wikipedia as a tool for contemporary history of science: A case study on CRISPR
Source: PLoS One. 2023 Sep 13;18(9):e0290827. doi: 10.1371/journal.pone.0290827 (PMC10499201; doi:10.1371/journal.pone.0290827)

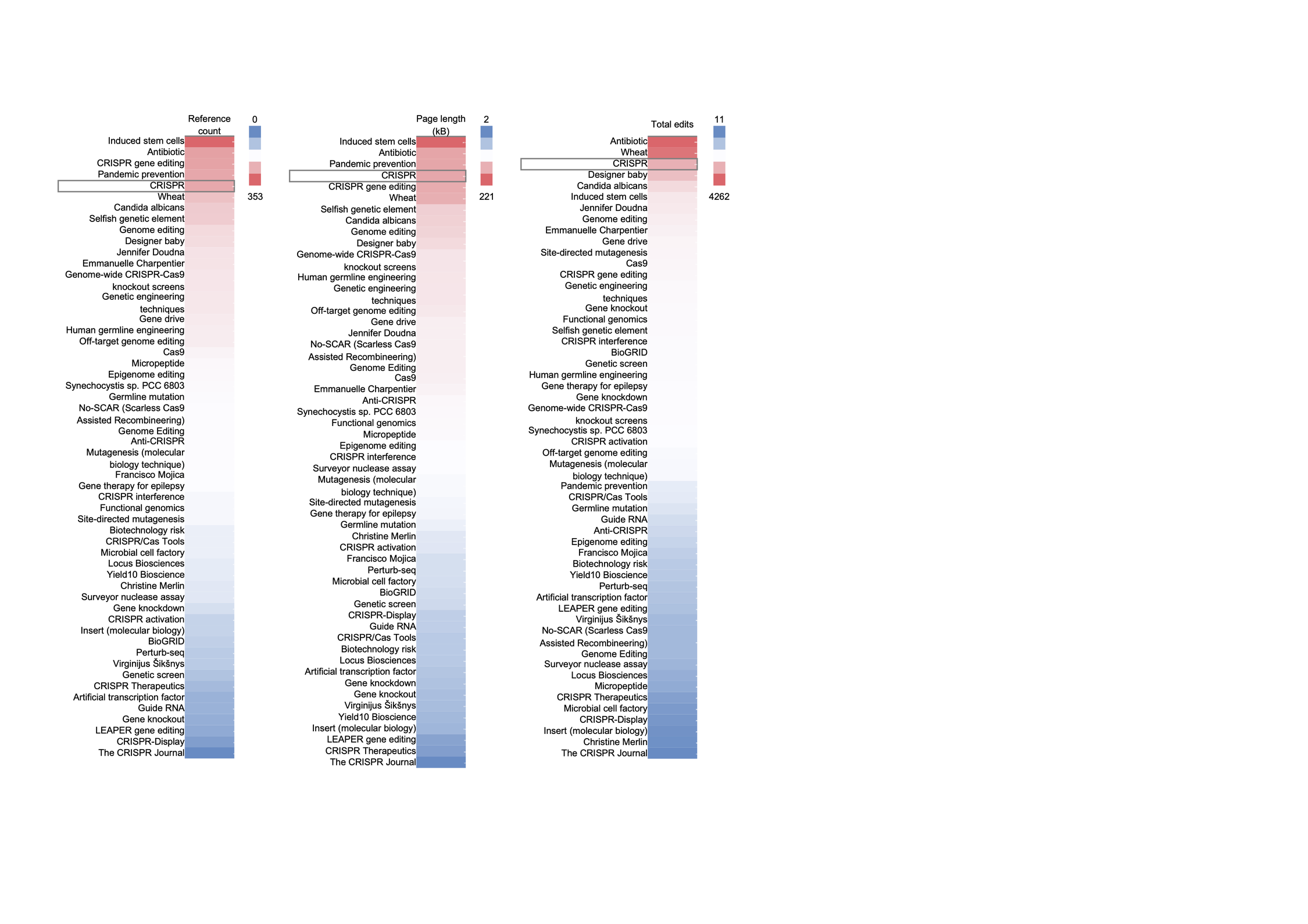

Supplement: S1 Fig — The articles included in the corpus, sorted by number of references, size in kilobytes (kB) and number of edits. “CRISPR”, highlighted, was among the top 5 articles of each category. (TIF) [file pone.0290827.s001.tif]

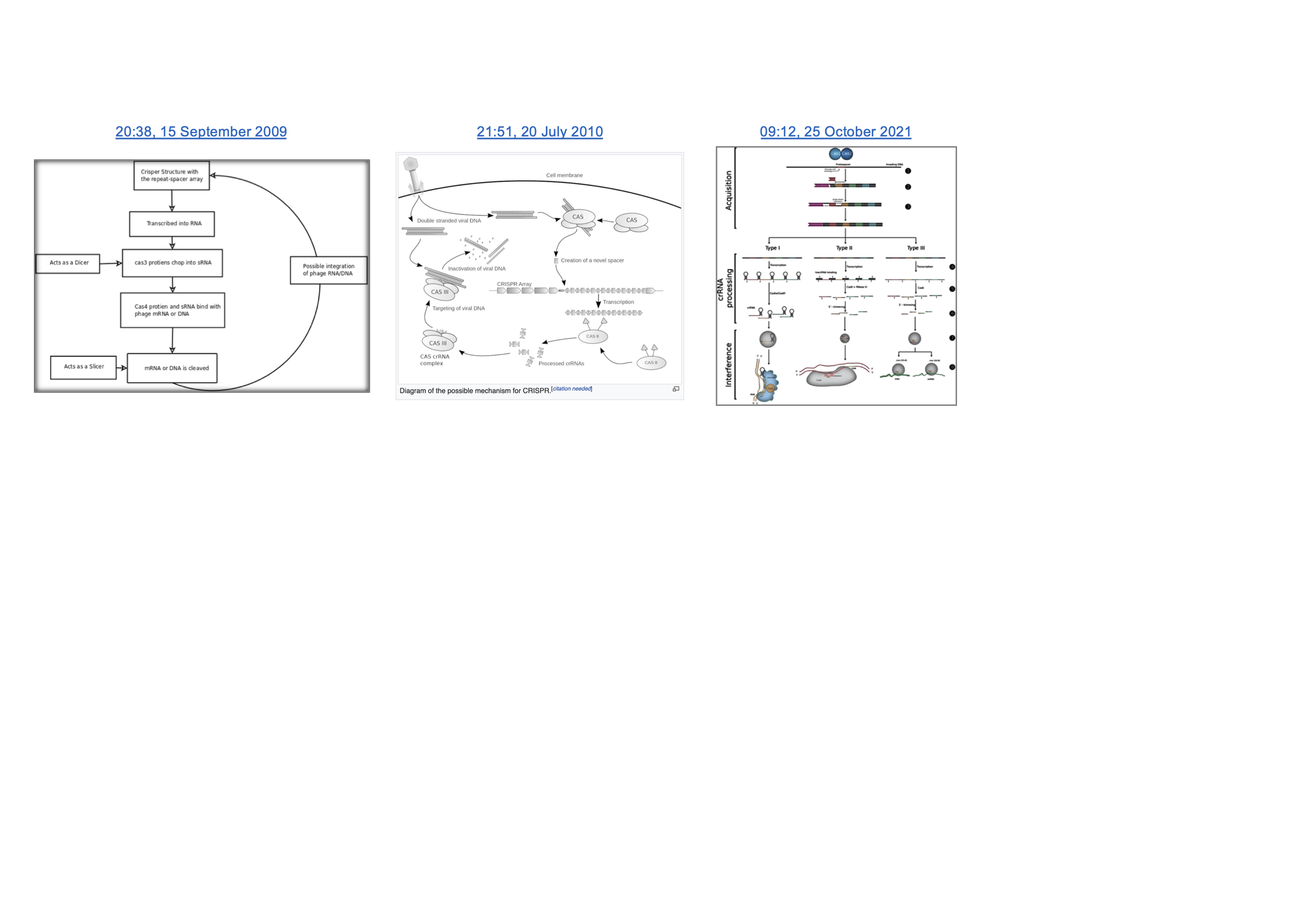

Supplement: S3 Fig — Shown are a selection of screen grabs from the CRISPR article, reflecting the evolution of Wikicommons graphics of CRISPR’s mechanism of action and key players. These are of different versions of the same illustration (A and B) and of a third illustration added later to the article. (TIF) [file pone.0290827.s003.tif]
